# The rare phenomenon of glomerulonephritis presentation in extra-nodal marginal zone lymphoma: summary of two cases and review of the literature

**DOI:** 10.1007/s00277-025-06245-w

**Published:** 2025-02-07

**Authors:** Michele D. Stanchina, Juan Pablo Alderuccio, Yiqin Zuo, Daniel Cassidy, Izidore S. Lossos

**Affiliations:** 1 Division of Hematology, Department of Medicine, Sylvester Comprehensive Cancer Center, University of Miami, 1475 NW 12th Ave (D8-4), Miami, FL 33136 USA; 2 Division of Pathology, Sylvester Comprehensive Cancer Center, University of Miami Miller School of Medicine, Miami, FL USA; 3 Department of Molecular and Cellular Pharmacology, University of Miami, Miami, FL USA

**Keywords:** Glomerulonephritis, Marginal zone lymphoma, MALT associated nephropathy, Extra-nodal marginal zone associated glomerulonephritis, Paraneoplastic glomerulonephritis

## Abstract

Paraneoplastic glomerulonephritis (GN) has been reported in non-Hodgkin lymphoma (NHL), Hodgkin Lymphoma (HL), and chronic lymphocytic leukemia (CLL). Despite descriptions in NHL, very few reports have been documented in marginal zone lymphoma (MZL). In this article, we review the literature of currently known cases of MZL-associated GN and we detail two cases of patients with extra-nodal MZL (EMZL) who both presented with renal failure, fluid overload, and proteinuria which were attributed to GN after a renal biopsy. We discuss the pathology of each renal biopsy in depth and how the diagnosis of GN was made, along with potential mechanisms of how EMZL led to GN. We also discuss the treatments each patient received and whether this led to the resolution of their GN. Both cases highlight the importance of maintaining a high index of suspicion for this paraneoplastic syndrome when patients present with signs or symptoms of renal failure, proteinuria or hematuria, or potential renal involvement on imaging. In these cases, a renal biopsy should be pursued to confirm the diagnosis, and treatment should be tailored accordingly.

## Introduction

Marginal Zone Lymphoma (MZL) is a distinct group of indolent mature B-cell lymphomas comprising approximately 7% of mature non-Hodgkin lymphomas (NHL), with 61% of cases being extranodal MZL (EMZL) of mucosa-associated lymphoid tissue (MALT). The average age of presentation is 60 years. EMZL can occur at any mucosal site, most commonly the stomach, ocular adnexa, lung, and salivary glands. However, renal involvement is less common [1, 2]. EMZL is an antigen-driven malignancy, initially associated with immune cross-reactions driven by chronic exposure to bacterial infections such as *Heliobacter pylori* (*H.pylori*) in the stomach, *Chlamydia psittaci* in the ocular adnexa, or autoimmune disorders such as Sjogren’s syndrome of the salivary glands or Hashimoto’s disease of the thyroid. Genetic aberrations, such as trisomies or translocations, can lead to tumorigenesis via *NF*-κβ pathway activation or NOTCH deregulation, which may sustain lymphoma growth independent of chronic stimulation [2]. While t(11;18), the first translocation identified in MALT lymphoma, remains the most common, trisomies of chromosome 3, 12, or 18 are detected in 20–30% of EMZL. The BIRC3 gene on 11q21, which inhibits apoptosis, is translocated to the MALT1 gene on chromosome 18q21, which encodes a crucial protein involved in B and T lymphocyte activation via the *NF*-κβ pathway. This translocation is most common in EMZL of the stomach and lung. There are additional translocations including t(1;14), t(14;18), t(3;14) which are less common and juxtapose IgH with BCL10, MALT1, and FOXP1 genes, respectively, resulting in their upregulation [3]. EMZL displays unique histologic and immunophenotypic features including a proliferation of small to medium-sized lymphocytes with frequent plasmacytic differentiation, that on immunohistochemistry (IHC) are positive for CD20, CD79a, PAX5 and negative for CD5, germinal center markers, CD23, cyclin D1 and SOX11 [3]. 

Lymphomas can impact renal function in multiple ways, including ureteral or renal vascular obstruction, direct renal parenchymal infiltration by the tumor, or tumor-related conditions such as hypercalcemia, tumor lysis, sepsis, and hemolysis [4]. Previous studies have reported renal injury in up to 10% of patients with NHL and chronic lymphocytic leukemia (CLL), however, clinically relevant involvement by low grade B-cell lymphomas such as MZL are less common [4–11]. Paraneoplastic glomerular diseases have also been reported, especially in Hodgkin lymphoma (HL) and CLL, but rarely in MZL [12–15]. Minimal change disease (MCD) and focal segmental glomerulosclerosis (FSGS) are frequently associated with HL, while membranoproliferative glomerulonephritis (MPGN) and membranous glomerulonephritis (MGN) are more commonly associated with CLL and NHL [16]. Here we describe two cases of paraneoplastic GN, with the goal of highlighting the difficulty in diagnosing this rare phenomenon, reviewing the available literature and better understanding the incidence and pathophysiology of this paraneoplastic process.

## Methods

We retrospectively reviewed a database of 772 patients diagnosed and treated with MZL seen at the University of Miami/Sylvester Comprehensive Cancer Center between June 1996 to November 2024 as previously reported [17–19]. This retrospective review study was approved by the local institutional review board (IRB) in accordance with the Declaration of Helsinki. We searched the database to identify patients with a clinical diagnosis of EMZL (608 patients) with diagnosis of GN or abnormal renal imaging that suggested involvement of the kidney by EMZL. Search terms such as “GN”, “renal”, or “kidney” were applied to identify patients. Overall, 11 patients were identified. The charts of these patients were reviewed, including clinician notes, laboratory values, and pathological results when available, to determine if the patient had abnormal kidney function based on blood tests or urinalysis to suggest a diagnosis of GN. Of these 11 patients, 4 patients were found to have GN, however, only two were attributed to EMZL-associated GN, while the other two were secondary to systemic lupus erythematosus (SLE). The remaining 7 cases had chronic kidney disease (CKD) defined as kidney damage or a decreased glomerular filtration rate (eGFR) of less than 60 mL/min lasting for at least three months but did not have any evidence of GN. In most cases, CKD was attributed to other co-morbidities such as long-standing hypertension or diabetes. One patient had MZL that transformed to Diffuse Large B-Cell Lymphoma and his renal failure was ultimately attributed to chemotherapy while another patient had concomitant renal cell carcinoma.

Additionally, we conducted a literature search using PubMed Medical Subject Headings (MeSH) including “glomerulonephritis”, “extra-nodal marginal zone lymphoma”, “nodal marginal zone lymphoma”, “splenic marginal zone lymphoma”, “renal-associated extra-nodal marginal zone lymphoma”, “membranoproliferative glomerulonephritis”, “non-Hodgkin lymphoma”, “extra-marginal zone lymphoma”, “marginal zone lymphoma”, “mucosa associated lymphoid tissue”, “MALT”, “MALT lymphoma”. Identified cases of MZL-associated GN in the literature were reviewed to determine the specific type of MZL, presenting symptoms, clinical course, treatment received, and overall incidence of MZL involving the kidney.

### Case #1

A 41-year-old African American male initially presented with one month of lower extremity edema, intermittent shortness of breath (SOB), fatigue, and foamy urine. On exam, he was found to be hypertensive with interstitial pulmonary edema on chest X-ray. Work up for congestive heart failure was unrevealing. Laboratory tests showed hypoproteinemia to 4.8 g/dL, hypoalbuminemia to 2.2 g/dL, along with proteinuria (> 600 mg/dL, urine protein to creatinine ratio 9.8 g/g), and hematuria (6 /HPF RBC) on the urinalysis. A 24-hour urine protein showed 4945 mg/24 hours of proteinuria. Free kappa and lambda were both elevated, 8.2 mg/dL and 21.89 mg/dL respectively, although the ratio was normal 0.375. Serum Protein Immunofixation (SPEP) did not show any M spike although the serum immunofixation (SIFE) did show an IgM-λ. The patient was also noted to have microcytic anemia (Hemoglobin 10 g/dL, MCV 75.4) and acute kidney injury (AKI) with serum BUN 31–43 mg/dL and creatinine 1.30–2.3 mg/dL (GFR 39–79 mL/min). Further work-up of the anemia showed iron 35 mcg/dL, TIBC 177 mcg/dL, ferritin 64 ng/mL, B12 584 pg/ML and folate of 4.7 ng/mL. He started folic acid supplementation and was given iron sucrose (Venofer) 200 mg injections for five days. Total cholesterol and low-density lipoprotein (LDL) were both elevated, with low high-density lipoprotein (HDL). Human immunodeficiency virus (HIV), Hepatitis B and Hepatitis C serologies were negative, along with antinuclear antibody (ANA), antineutrophil cytoplasmic antibodies (ANCA), and antiphospholipid A2 receptor antibody (PLA2R), which is strongly associated with idiopathic GN.

**Fig. 1:**
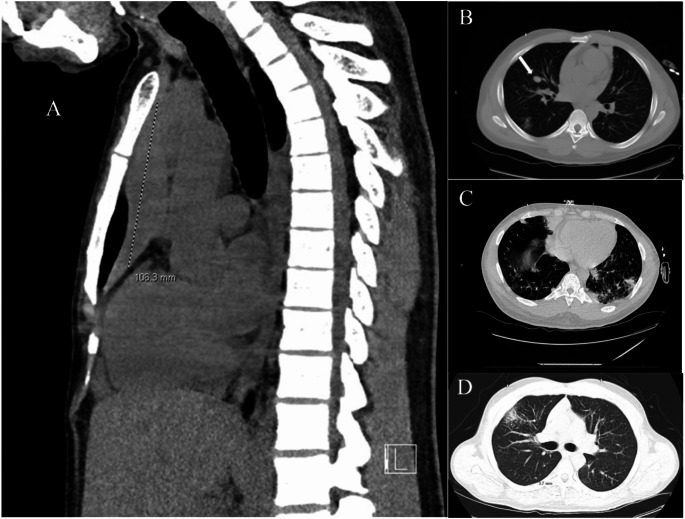
CT Chest at presentation. (**A**) A 4.7 × 3.0 × 10 cm homogeneous soft tissue density lesion in the anterior mediastinum; (**B**) A 1.8 cm nodule in the right middle lung lobe (white arrow); (**C** and **D**) Focal, patchy opacities

On computed tomography (CT) imaging, the patient had bilateral multifocal centrilobular alveolar opacities and a lingular subsegmental consolidation which raised concerns of infection. Additionally, he had 4.7 × 3.0 × 10 cm homogeneous soft tissue density lesion in the anterior mediastinum, with additional lung nodules including a 1.8 cm nodule in the right middle lobe and enlarged lymph nodes in the right pericardiophrenic region measuring up to 3.3 × 1.4 cm, which were concerning for malignancy (Fig. 1). The patient underwent an extensive infectious work-up including testing for tuberculosis, legionella, COVID, influenza, respiratory viruses which were all negative. He underwent a bronchoalveolar lavage which was positive for Klebsiella Pneumoniae and was treated with Cefdinir 300 mg twice daily for five days.

However, there remained concern for malignancy as no biopsy was completed. Due to proteinuria, a renal ultrasound (US) was completed and while it did not show hydronephrosis it revealed a hypoechoic structure within the left kidney measuring up to 2.3 cm. Due to the proteinuria and high concern for malignancy, a renal biopsy was pursued. The biopsy was a limited sample with eleven glomeruli analyzed by all study modalities. The biopsy consisted of one piece of corticomedullary junction containing three glomeruli, none of which were globally sclerosed. By light microscopy (LM), the mesangial matrix and glomerular basement membranes (BM) were unremarkable. There were no adhesions, crescents, fibrin, or necrosis in Bowman’s spaces and no evidence of segmental sclerosis, hyalinosis, thrombi, or endocapillary hypercellularity of glomerular tufts. There was minimal interstitial fibrosis and tubular atrophy, occasional proteinaceous casts without intratubular crystals or polarizable material and no overt acute tubular injury. There was 1–2+, very segmental granular mesangial staining by immunofluorescence (IF) for IgG, kappa, and lambda light chain but no glomerular staining for IgA, IgM, C3, or C1q. Casts were stained 2–3 + for IgM, 3 + for IgA, C3, kappa and lambda light chains. The main finding was extensive foot process effacement (80–90%) by electron microscopy (EM) suggesting either MCD or FSGS. The glomerular BM were moderately thickened with an average of 640 nm (average GBM thickness in adult men is 370 nm). The corticomedullary junction, the area where FSGS usually first develops, was sampled but there was minimal sclerosis; however, FSGS was not ruled out as < 25 glomeruli were examined. The patient was discharged on diuretics, atorvastatin, and anti-hypertensives without complete clarity of the cause of his renal dysfunction. While a trial of steroids was considered, it was recommended that the patient first undergoes additional work-up for a possible malignancy. Unfortunately, after discharge from the hospital, the patient was re-admitted shortly afterwards with COVID pneumonia, and the decision was made to hold off on evaluation of his renal dysfunction and evaluation of malignancy. Of note, during this admission he had microscopic hematuria and 24-hour urine protein of 12,948 mg/24hours.

**Fig. 2:**
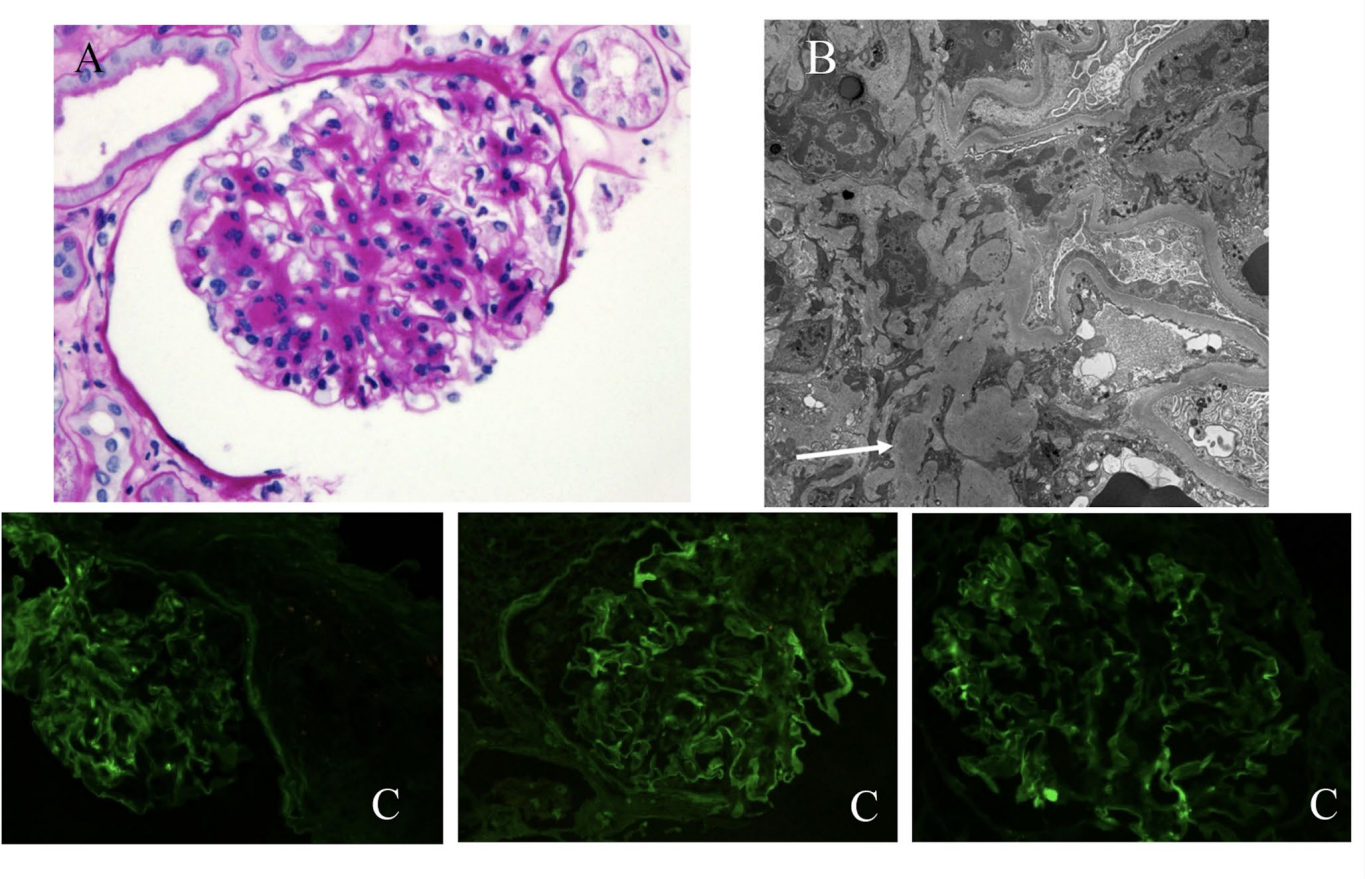
Kidney Biopsy. (**A**) Light Microscopy of kidney biopsy 400X: There is focal, segmental, mild to moderate, increase in mesangial matrix and cellularity (Periodic acid-Schiff stain, 400x); (**B**) Electron microscopy 1200X: Visualization of electron-dense deposits (mesangial deposits- white arrow); (**C**) Immunofluorescence of kidney biopsy 400X: 1–2+, focal, segmental, granular mesangial staining for IgG (left), κ (middle) and λ (right) light chains

The patient was lost to follow-up, and it wasn’t until four months later that he underwent a repeat renal biopsy of the right kidney due to limited glomeruli visualization on the initial biopsy. By LM, there were 21 glomeruli without global or segmental sclerosis and minimal interstitial fibrosis. There was focal, segmental, mild to moderate mesangial expansion by eosinophilic deposits and mesangial cellularity (Fig. 2A). Congo red special stain was negative. By EM there were occasional mesangial deposits with non-specific fibrils (Fig. 2B). By IF, on a scale of 0–3+, there was 1–2+, focal, segmental, granular mesangial staining for IgG, kappa and lambda light chains (Fig. 2C). There was no glomerular staining for IgA, IgM, C3, or C1q. There was 3 + vascular staining for C3 and 3 + tubular protein droplet staining for albumin. Overall, the finding was most consistent with mesangial proliferative GN based on expert renal pathological review and were not consistent with MPGN characterized by GBM with splitting/tram-track/ double contours, frequently with endocapillary hypercellularity that were not seen in the biopsy.

**Fig. 3:**
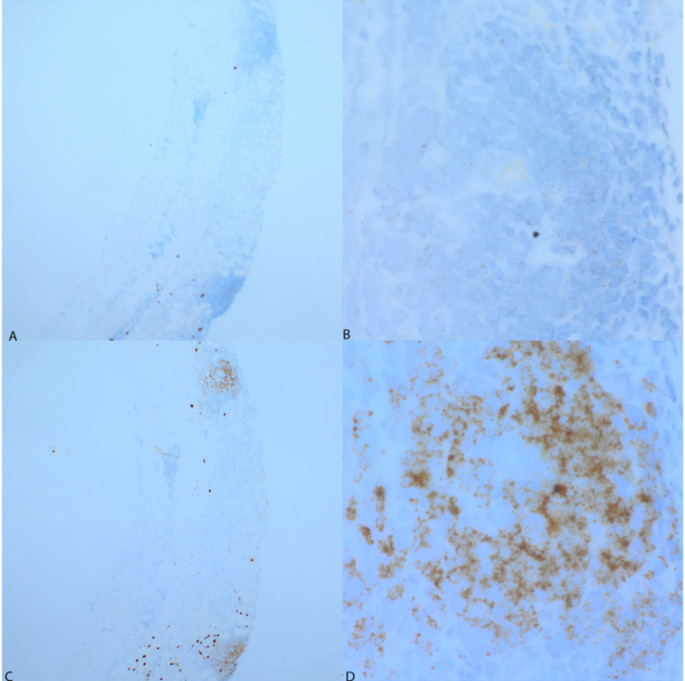
Mononuclear inflammatory infiltrate from the renal parenchyma. RNA chromogenic in-situ hybridization (ISH) for kappa (**A**, 40X and **B**, 400X) and lambda (λ) (**C**, 40X and **D**, 400X) light chains highlight a λ predominant population of B-cells and plasma cells

In addition, a mononuclear inflammatory infiltrate was also observed in the renal parenchyma near the capsular region. The biopsy was reviewed by hematopathology with findings of cytologic atypia, relative predominance of B-cells, and λ predominance that was seen in the B-cells and plasma cells raising the possibility of an underlying low-grade B-cell lymphoma with plasmacytic differentiation. IHC disclosed CD20 positive B cells that were negative for CD10 and CD5; k and λ light chain by RNA chromogenic in-situ hybridization (ISH) highlighted a λ predominant population of B-cells and plasma cells (Fig. 3). BCL-2 highlighted scattered T-cells and B-cells, overall consistent with an atypical lymphoplasmacytic infiltrate involving renal parenchyma. SPEP had two small paraprotein peaks, IgM λ and IgG λ, each about 0.1 g/dL, and SIFE showed a monoclonal IgG- λ and monoclonal IgM-λ, although both the serum IgM levels and serum free light chain (FLC) ratio were normal. Due to worsening edema and persistent proteinuria (24-hour urine protein of 15,384 mg/24 hours), he was started on intravenous methylprednisolone at a dose of 500 mg daily for three days, followed by a single dose of rituximab at 375 mg/m² with improvement in edema and 24-hour proteinuria to 2760 mg/24 hours.

The patient underwent bronchoscopy with multiple transbronchial biopsies which demonstrated atypical lymphoid aggregates present in each biopsy in a diffuse and nodular, peribronchial distribution and composed of predominantly small lymphoid cells with variable monocytoid and lymphoplasmacytic morphologic features. By IHC lymphoma cells were positive for CD20 and BCL2 and negative for CD3, CD5, and CD10. B cells were lambda light chain restricted by kappa and lambda RNA ISH studies. Flow cytometry studies from transbronchial biopsies and peripheral blood demonstrated the same B cell population.

**Fig. 4:**
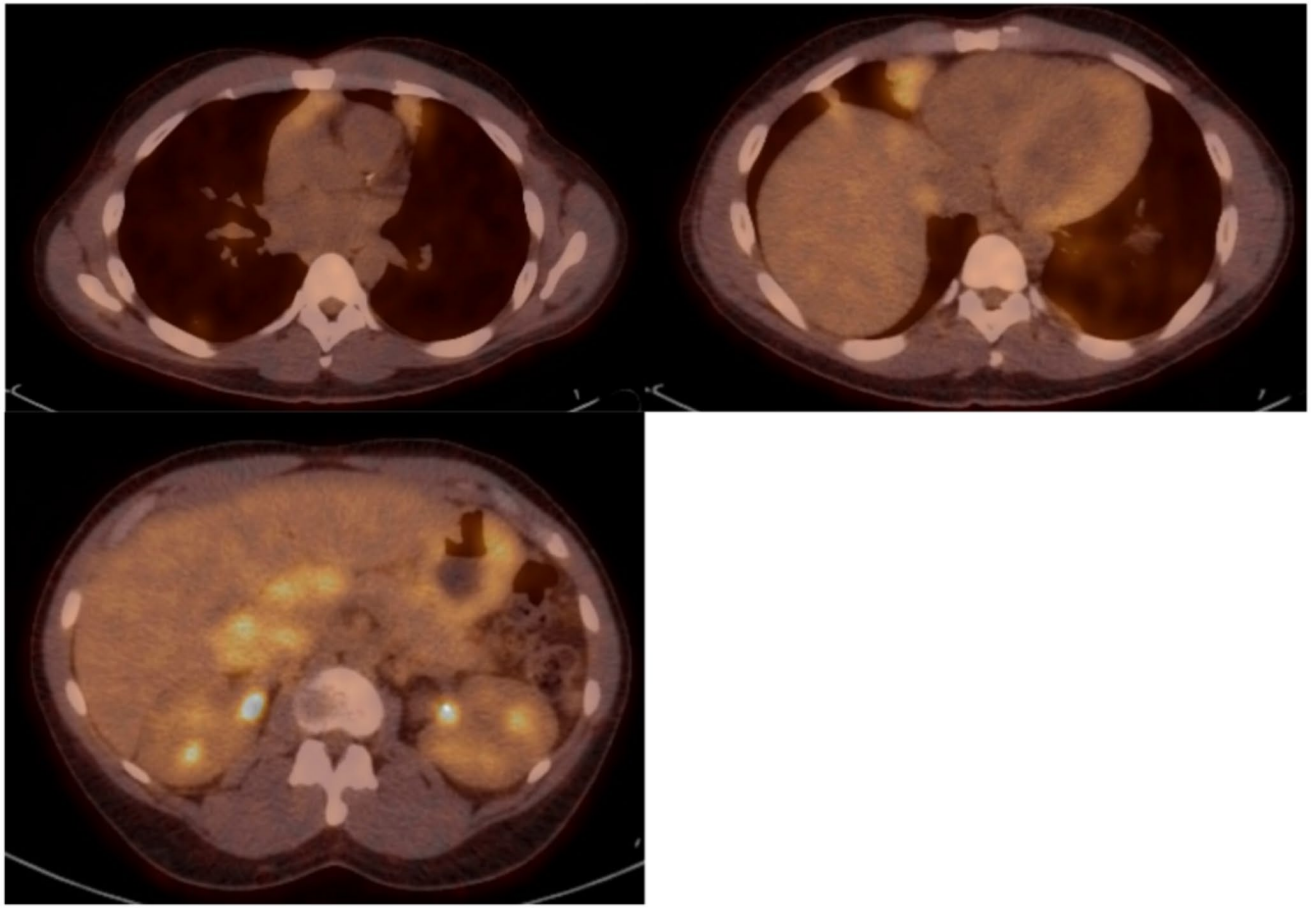
PET CT-study. A large anterior mediastinal mass with extension to the thoracic inlet, mediastinal lymph nodes, liver lesions, and periportal lymph nodes near the head of the pancreas were demonstrated

The patient underwent full staging with 18 F-2-deoxy-2-fluoro-d-glucose-labelled positron emission tomography (PET-CT) which again showed a large anterior mediastinal mass with extension to the thoracic inlet, measuring 9.9 × 5.4 × 2.5 cm with a standardized uptake value (SUV) of 3.2, compared to liver and mediastinal SUV uptakes of uptake 2.6 and 1.6, respectively. In addition, multiple FDG avid lesions in the liver were observed, including one 6.9 × 6.9 cm mass near the inferior edge of the liver, and another near the small bowel, SUV 8.7, measuring approximately 9.6 × 7.3 cm, periportal lymph nodes inseparable from the head of the pancreas measuring approximately 7.4 × 4.4 cm, retroperitoneal and mesentery lymphadenopathy, and thickening of the stomach with increased SUV to 4.4 (Fig. 4). Bone marrow biopsy demonstrated involvement by EMZL, comprising approximately 20% of marrow cellularity, leading to the diagnosis of stage IV EMZL. Further workup showed hemoglobin 9.6 g/dL, uric acid 9.2 mg/dL, erythrocyte sedimentation rate (ESR) 90 mm/hour, beta 2 microglobulin 4.31 mg/L and normal lactate dehydrogenase (LDH).

**Fig. 5:**
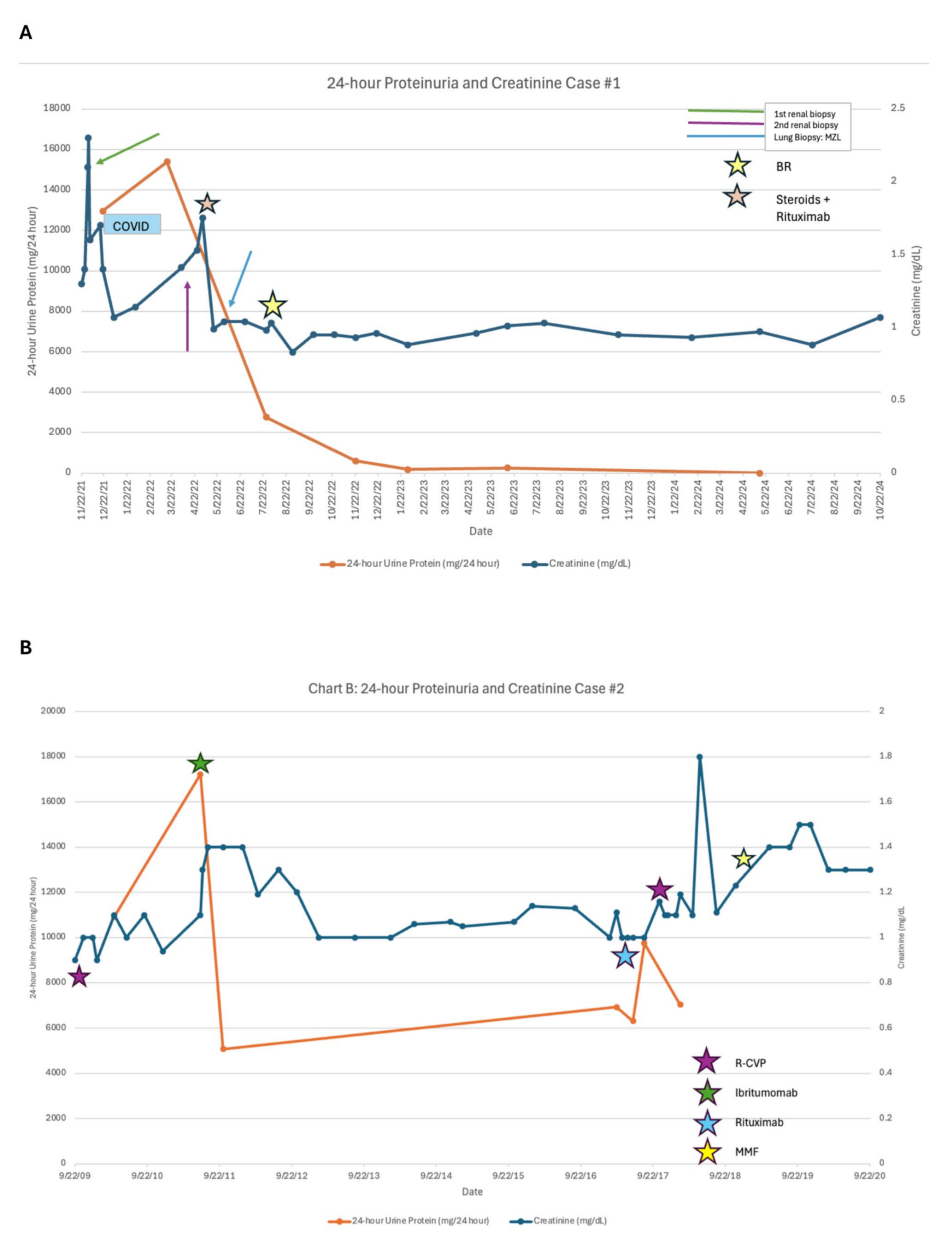
Timeline of clinical course. (**A**) 24-hour proteinuria and creatinine of patient 1 over clinical course. Green arrow represents first renal biopsy, purple arrow represents second renal biopsy and blue arrow is lung biopsy that diagnosed Marginal Zone Lymphoma (MZL). The stars represent when the patient received treatment. The light orange star indicates when the patient received steroids and rituximab. The yellow star represents when the patient received chemoimmunotherapy with Bendamustine and Rituximab (BR) (**B**) 24-hour proteinuria and creatinine of patient 2 over clinical course. The stars represent when the patient received treatment. The purple star represents Rituximab-cyclophosphamide, vincristine, prednisone (R-CVP), although vincristine was omitted during the second administration. The green star is Ibritumomab tiuxetan, blue star is Rituximab single agent and yellow star is mycophenolate mofetil (MMF)

The patient was diagnosed with stage IV EMZL with multi-mucosal site presentation. A prior large retrospective study of EMZL patients presenting with multiple mucosal site (MMS) involvement, involving two or more organs independent of the spleen and bone marrow involvement, demonstrated shorter progression-free survival (PFS) (HR: 5.39, *P* < 0.001) with a median PFS of 1.7 vs. 13.2 years and shorter overall survival (OS) (HR: 4.44, *P* < 0.001) with 10-year OS of 40.5% (95% CI: 20.7–59.5%) vs. 81.1% (95% CI: 75.1–85.8%) for patients with and without MMS, respectively which is also associated with higher risk of transformation [20, 21]. Thus, the patient was started on treatment with bendamustine-rituximab (BR) for six cycles, achieving a complete response (CR) after cycle 4, with complete resolution of his creatinine to baseline, although his 24-hours urine protein remained elevated at 595 mg/24 hours. The patient remains in CR 20-months post-treatment with significant resolution of his proteinuria with the last known value of 250 mg/24 hours. A timeline of his renal failure, associated proteinuria and treatment course can be found in Fig. 5A.

### Case #2

A 63-year-old Caucasian male with hypertension on a beta-blocker, calcium channel blocker and ACE-inhibitor initially presented with pulmonary nodules, nephrotic range proteinuria, and a normal BUN and creatinine. Initial work-up for HIV, hepatitis B and C, and rheumatological testing including ANCA, anti-smith, anti-RNP, anti-SSA/SSB and anti-scleroderma 70 were all negative. Pulmonary nodules were biopsied at an outside hospital showing diffuse aggregates of small atypical lymphoid cells with monocytoid appearance and lymphoplasmacytic features. By IHC lymphoma cells were positive for CD20 and BCL2 and negative for CD5, and CD10 leading to stage II EMZL diagnosis. He underwent a renal biopsy and was diagnosed with stage 2–3 MGN, likely secondary to malignancy, given the histological findings of increased mesangial cellularity, and scattered mesangial electron dense deposits. The renal biopsy had 14 glomeruli available for assessment, four of which showed global sclerosis. The remaining glomeruli showed mild increase in mesangial cellularity and mild expansion of the mesangial matrix by LM. The capillary walls were thickened, with frequent holes and spikes identified by silver stain. There was no endocapillary proliferation or crescents. The interstitium showed mild, mixed inflammatory infiltrate along with interstitial fibrosis involving approximately 10% of the biopsy. There were several tubules noted to have proteinaceous casts. Arterioles and interlobular arteries showed mild thickening and fibrosis. Five glomeruli were available for IF review, none of which showed global sclerosis. IF showed 3 + diffuse, granular capillary loop and mesangial IgG, IgA, and κ light chain staining within the glomeruli, along with similar staining patterns within the tubular BM and vascular walls. There was a 1 + capillary loop and mesangial granular staining within the glomeruli for IgM. Lamba light chains showed 1 + staining within the glomeruli and tubular casts. There was a C3 (2+), and C1q (1+) staining in similar pattern as IgG, and a 3 + granular staining for C3 in tubular BM and 2 + in vascular walls. On EM, the glomerular BM was thickened and contained numerous subepithelial and intramembranous electron dense deposits. The epithelial cells showed cytoplasmic vacuoles, microvillous transformation, and near complete foot process effacement. The mesangial cells were expanded and small electron dense deposits were also identified.

Given his stage II MZL with paraneoplastic GN, the patient completed 6 cycles of rituximab, cyclophosphamide, vincristine, prednisone (R-CVP) in February 2010 with reduction in proteinuria from 500 mg/dL on urinalysis to 150 mg/dL, and had stable, low FDG-avid pulmonary nodules. His fatigue and SOB persisted post-treatment, however, this was thought to be attributed to newly diagnosed emphysema. He continued to have debilitating fatigue, and elevated blood pressure with heavy proteinuria measuring 10,925 mg/24 hours. PET-CT in December 2010 and May 2011 showed increased FDG uptake in the pulmonary nodules. Bone marrow biopsy was negative for lymphoma. Proteinuria continued to worsen with 17,226 mg of protein/24 hours. Given the level of proteinuria, and concern for EMZL progression, he was treated with Ibritumomab tiuxetan, a monoclonal antibody radioimmunotherapy, in July 2011. Proteinuria improved to 5076 mg/24 hours but did not resolve completely. He continued to have worsening edema and SOB requiring oxygen; however, he was found to have a new left bundle branch block, atrial fibrillation, depressed ejection fraction of 35%, and coronary artery disease requiring stent placement. Metolazone was added to his diuretic regimen which significantly improved his symptoms. PET-CT showed stable disease in the lungs, but increased low-level FDG activity in the stomach. Esophagogastroduodenoscopy (EGD) showed gastritis but no clear evidence of lymphomatous involvement. With longer follow up following Ibritumomab tiuxetan the patient had significant improvement in his blood pressure and edema and PET-CT in 2013 showed CR. The patient remained in CR until February 2017 when he started to have night sweats and cough. LDH and ESR were mildly elevated to 264 U/L and 20 mm/hour, respectively. BUN was 17 mg/dL and creatinine was 1 mg/dL; however, the patient had worsening nephrotic range proteinuria (24-hour urine protein of 6925 mg/24 hours). Urine analysis showed free κ light chain of 22.9 mg/L, λ light chain of 5.9 mg/L, and a κ/ λ ratio of 3.88. PLA2R, which is usually positive in patients who have primary MGN, was negative, and nephrology attributed the proteinuria to secondary MGN, making it imperative to rule out lymphoma recurrence. PET-CT demonstrated worsening of bilateral FDG avid pulmonary nodules concerning for recurrent lymphoma, and the patient was started on rituximab weekly for four doses which he completed in May 2017. Unfortunately, there was minimal response, and 24-hour protein increased to 9750 mg/24 hours by August 2017. He was started on R-CVP and completed 5 cycles, with omission of vincristine due to underlying neuropathy. Despite the improvement in EMZL of the lung, the GN persisted as confirmed on repeated biopsy and continuously elevated proteinuria of 7040 mg/24 hours in February 2018 at the end of therapy. He was started on mycophenolate (MMF), without improvement. His last known creatinine was 1.41 mg/dL, and urine protein/Cr ratio was 5769 mg/g creatinine in February 2022 after which he was lost to follow up. A timeline of his renal failure, associated proteinuria and treatment course can be found in Fig. 5B.

## Discussion

Lymphomatous involvement of the kidneys by MZL is more common than previously suggested [3, 6, 13, 16, 22]; however, paraneoplastic glomerular disease is a rare phenomenon with few case reports in the literature showing associations between EMZL and GN [12, 14, 16, 23–25]. For example, in a review of 700 patients with NHL and CLL, 83 were found to have renal failure, but only 5 were due to GN, none of which were associated with EMZL [26]. In our database of 608 patients with EMZL, we found two cases of EMZL associated GN, amounting to 0.3% of our cases. We performed a literature search for all known cases of MZL and paraneoplastic GN, and detected an additional 15 cases, including 12 EMZL patients, summarized in Table 1.

**Table 1:** Reported cases of EMZL and Glomerulonephritis

Type of Lymphoma	Age of Patient	Sex	Renal Pathology	Presenting Symptoms	Treatment	Outcome	Reference
Low grade B-cell NHL	64	M	MPGN, Type I	HTN, nephritic/nephrotic syndrome, AKI	CVP	CR + resolution of symptoms	Alshayeb et al.[12]
Gastric MALT	62	M	IgA Nephropathy	HTN, hematuria, anorexia, fatigue, weight loss, anemia, proteinuria, AKI	Chlorambucil	CR + resolution of symptoms	Mak et al.[24]
Gastric MALT	52	M	Unknown	AKI, nephritic/nephrotic syndrome, anemia	Rituximab + Steroids	CR + resolution of symptoms	Li et al. [16]
Gastric MALT	58	F	Exudative GN	AKI, LAD, perioral edema, proteinuria, microscopic hematuria	RT for gastric MALT; RCHOP for EMZL	CR + resolution of symptoms	Yeo et al. [14]
Splenic MZL	72	M	Unknown	AKI, proteinuria, pancytopenia, decreased complement, hyper-gammaglobulinemia	Steroids	Resolution of complement and gamma-globulins; persistent microscopic hematuria and minor proteinuria	Adamidis et el. [4]
Splenic MZL	50	M	MPGN	AKI on CKD, proteinuria, anemia, HSM	HD; no treatment for SMZL	HD dependent	Chelioti et al. [31]
Renal MZL	58	M	MCD	HLD, edema, abdominal distension, weight gain, proteinuria	R-CVP	CR + resolution of symptoms	Kasmani et al. [22]
Renal MZL	68	M	MPGN	AKI, hematuria, nephrotic proteinuria, fatigue	Steroids	Unknown	Stokes et al. [13]
Renal MZL	72	F	MPGN	AKI, nephrotic proteinuria, peripheral edema	Steroids	CR + resolution of symptoms	Stokes et al. [13]
EMZL	66	F	MPGN with cryoglobulins	AKI, proteinuria, Ig-M kappa, low complement	Rituximab, Steroids, PLEX	CR + resolution of symptoms	Akiyama et al. [23]
EMZL	70	M	Mesangial proliferative GN	AKI, petechial rash, elevated cryoglobulins	Rituximab + Steroids	CR + resolution of symptoms	Yamada et al. [32]
EMZL	61	M	MPGN	AKI, rash, elevated cryoglobulins, low complement	Rituximab + Steroids	CR + resolution of symptoms	Yamada et al. [32]
MALT, Unspecified	Unknown	Unknown	AL	Unknown	RCHOP	SD MALT; PR AL	Nie et al. [25]
MALT, Unspecified	Unknown	Unknown	IgA nephropathy	Unknown	BR	CR + resolution of symptoms	Nie et al. [25]
MALT, Unspecified	Unknown	Unknown	GN with cryoglobulins	Unknown	None	Unknown	Nie et al. [25]

AL: amyloid light chain; AKI: acute kidney injury; BR: Bendamustine + Rituximab; CKD: chronic kidney disease; CR: complete response; EMZL: Extra-nodal marginal zone lymphoma; CVP: cyclophosphamide, vincristine, prednisone; F: female; GN: glomerulonephritis; HD: hemodialysis; HLD: hyperlipidemia; HSM: hepatosplenomegaly; HTN: hypertension; LAD: lymphadenopathy; M: male; MALT: mucosa associated lymphoid tumor; MCD: minimal change disease; MPGN: membranoproliferative glomerulonephritis; MZL: marginal zone lymphoma; NHL: Non-Hodgkin lymphoma; PR: partial response; PLEX: plasma exchange; RCHOP: Rituximab, cyclophosphamide, hydroxydaunorubicin, oncovin, prednisone; RT: radiotherapy; SD: stable disease; SML: Splenic Marginal Zone Lymphoma

Both EMZL involving the kidney and paraneoplastic GN secondary to a MALT lymphoma have variable treatment outcomes and can be associated with multiple relapses, but generally have good prognosis due to the underlying indolent nature of the disease [3, 10, 15]. In our personal experience, patients with isolated kidney involvement by EMZL can be treated with radiation, preserving kidney function and leading to long-term responses. Upon diagnosis of EMZL, it is imperative to understand which extra-nodal areas are involved. If there is concern for renal involvement on imaging, we suggest it would be beneficial to do a thorough baseline work-up including analysis of creatinine and GFR, SPEP, SIFE and free kappa and lambda light chains, along with a urinalysis to look for proteinuria and hematuria. If there is a high suspicion for GN, we would recommend a renal biopsy to confirm the diagnosis, improve the recognition of EMZL-associated GN, and enhance the understanding of the pathogenesis of this paraneoplastic syndrome.

The pathogenesis of paraneoplastic glomerular disease is incompletely understood; however, there are multitude of cases suggesting a relationship between lymphoid proliferation, immunological abnormalities, and glomerular involvement. Proposed mechanisms include B-cell clone secretion of immunoglobulins or cryoglobulins, tumor antigen insertion into glomeruli, and altered T-cell function [4, 5, 16]. Conversely, glomerular disease from an underlying inflammatory disorder such as SLE or sarcoidosis could be a predisposing factor for MZL [3, 7, 13, 27]. Patient 1 presented with nephrotic syndrome and had a small abnormal B cell clone related to the EMZL that infiltrated the kidney, which upregulated mesangial cell activity and led to mesangial proliferative GN. Mesangial cells in the kidney are a type of mesenchymal stromal cell (MSC) which includes fibroblasts, pericytes and vascular smooth muscle cells. MSC are involved in regulating tissue formation and angiogenesis, organizing the structure of the glomerulus, maintaining endothelial and podocyte homeostasis by controlling cell migration and proliferation, regulating a variety of processes including immunity and inflammation, and potentially contributing to the repair of podocytes after injury [28]. It has been proposed that mesangial cells may act similarly to other stromal cells, initiating an innate immune response in response to renal injury, releasing chemokines to direct immune cell recruitment. In turn, a maladaptive mesangial cell response could prevent resolution of tissue injury, increase rates of fibrosis, and drive underlying kidney dysfunction [29]. Following treatment of the patient’s EMZL and resolution of the underlying clone, the maladaptive mesangial response resolved and the patient’s creatinine returned to its baseline. This is consistent with EMZL in other anatomic locations where inflammatory conditions predispose the patient to lymphomagenesis but recede with resolution of the inflammatory response.

Case 2 further emphasizes the complexity of the relationship between EMZL and GN. This patient presented with pulmonary nodules which were found to be EMZL, and proteinuria which easily could have been attributed to AKI caused by common underlying conditions such as arteriosclerosis due to hypertension; however, the renal biopsy showed MGN, highlighting the necessity of obtaining an adequate renal biopsy with ample glomeruli to ensure an accurate diagnosis. MGN is less common than MPGN in NHL. Features that are atypical of MGN such as segmental mesangial proliferation, subepithelial deposits of fibrillary material and monoclonal IgG FLC-κ deposits have been reported in CLL and NHL [5]. Similar to MPGN, monoclonal proteins produced by B cells may be involved in the pathogenesis of nephropathy in paraneoplastic associated MGN. The patient was treated initially with R-CVP, and then Ibritumomab tiuxetan on relapse, with improvement of his proteinuria and pulmonary nodules both times. However, on his third relapse, re-treatment with R-CVP (omitting vincristine) improved his lung lesions, but not his proteinuria.

The majority of cases in the literature summarized in Table 1 had complete resolution of both their EMZL/MZL and their GN following lymphoma treatment, suggesting that paraneoplastic GN frequently responds to treatment of the underlying malignancy. This is line with patients with oncological cancers where the majority of patients achieve remission of nephrotic syndrome following tumor resection or chemotherapy [30]. Failure to treat the underlying malignancy can lead to worsening renal disease, as was evident with one report of a patient with splenic MZL and MPGN who never received treatment for his lymphoma, and ultimately ended up on hemodialysis [31]. However, there may be a subset of patients where remission of the malignancy is not correlated with the resolution of the renal dysfunction, as was the case with patient 2. While resolution of GN after successful systemic treatment of EMZL supports the concept of a paraneoplastic process, prior case series have also reported that renal improvement is not always achieved despite resolution of the EMZL, although the underlying mechanisms remain unclear [15]. One potential explanation for this is related to the depth of the response to treatment. Since most disseminated EMZL patients have non-curable disease with recurrent relapses, patients with resolution of GN post treatment might achieve a deeper response with less residual disease than patients with persistent GN, despite the absence of radiographically detectable disease in both scenarios. It is also possible that with multiple relapses the kidney undergoes structural changes from immune complex deposition which leads to irreversible damage. Further research is needed in this area to better understand the mechanism of renal damage in this scenario and it might be beneficial to ensure patients have a repeat renal biopsy to ensure the resolution of the paraneoplastic syndrome. Ideally, the patient in Case 2 would have undergone a repeat renal biopsy but unfortunately, he was lost to follow-up.

In conclusion, we posit that EMZL-associated GN is an underrecognized phenomenon, as proteinuria and AKI are more commonly attributed to other common medical co-morbidities such as chronic kidney disease, diabetes mellitus and hypertension, thus fewer renal biopsies are pursued. It is imperative that paraneoplastic glomerular diseases be considered in the differential for proteinuria in the presence of lymphomas, including EMZL. The pathological type of GN vary, although MPGN is most commonly reported. A multi-disciplinary approach is necessary for proper diagnosis and treatment. Patients with EMZL and GN should also continue long-term follow up with close attention to creatinine and proteinuria to evaluate for EMZL relapse and long-term renal sequalae.

## Data Availability

No datasets were generated or analysed during the current study.
